# Comparative Assessment of Proximal Humeral Bone Density Using CT Osteoabsorptiometry, Bone Microarchitecture Analysis, and a HU-Based Calibration Method: A CT and Micro-CT Study in Elderly Body Donors (65–86 Years)

**DOI:** 10.3390/diagnostics16050756

**Published:** 2026-03-03

**Authors:** Susanne Strasser, Lorenz Adam, Lukas Kampik, Rohit Arora, Johannes Dominikus Pallua

**Affiliations:** Department of Orthopaedics and Traumatology, Medical University Innsbruck, 6020 Innsbruck, Austria; susanne.kathrein@i-med.ac.at (S.S.); lorenz.adam@hotmail.com (L.A.); lukas.kampik@i-med.ac.at (L.K.); rohit.arora@i-med.ac.at (R.A.)

**Keywords:** proximal humerus, bone mineral density, CT-osteoabsorptiometry, micro-CT, bone microarchitecture, osteoporosis, diagnostic imaging

## Abstract

**Background**: Local bone quality of the proximal humerus is a key determinant of fracture risk and implant stability in osteoporotic bone. Beyond established HU-based calibration, CT-osteoabsorptiometry (CT-OAM)-derived indices and microarchitecture-oriented workflows warrant systematic cross-modality evaluation. **Methods**: Twelve proximal humeral heads from six body donors (age 65–86 years; bilateral specimens) were analyzed using paired clinical CT and high-resolution micro-CT. Bone quality was quantified by (i) a HU-calibrated cancellous vBMD method (Krappinger et al.), (ii) a CT-OAM-inspired workflow reporting an ROI-averaged mean-intensity index in arbitrary units (a.u.), and (iii) a calibrated Bone Microarchitecture Analysis (BMA) workflow in Analyze 15.0. Paired tests, linear regression, and repeated-measures ANOVA after z-standardization were applied. **Results**: HU calibration yielded a mean trabecular vBMD of 114.37 ± 35.15 mg/cm^3^ on clinical CT. The BMA workflow produced higher CT-based values (207.37 ± 23.78 mg/cm^3^, *p* < 0.001) and markedly higher micro-CT values (469.34 ± 30.99 a.u.), indicating a systematic level shift between calibration frameworks. The CT-OAM index averaged 166.94 ± 40.12 a.u. on clinical CT and 455.89 ± 132.63 a.u. on micro-CT. Cross-modality agreement was very strong for CT-OAM (R^2^ = 0.888) and moderate for BMA (R^2^ = 0.502). After z-standardization, no significant differences were detected between the three CT-based approaches. **Conclusions**: A CT-OAM-inspired ROI-mean index and a BMA-based workflow provide complementary, transferable readouts of proximal humeral bone quality across clinical CT and micro-CT, with stronger cross-modality rank consistency for CT-OAM. Absolute density values differ systematically between calibration frameworks and should not be interpreted as directly interchangeable. These approaches support opportunistic, site-specific bone quality assessment from routine CT, but require prospective validation against fixation-related outcomes and robust scanner-independent standardization.

## 1. Introduction

Bone mineral density (BMD) and bone microarchitecture are fundamental determinants of skeletal strength and fracture resistance. Osteoporosis, characterized by reduced bone mass and structural deterioration of bone tissue, represents a major public health challenge and is associated with a substantially increased risk of fragility fractures and implant-related complications in orthopedic surgery [1,2,3,4]. Beyond systemic bone loss, site-specific bone quality has been shown to critically influence fracture patterns, fixation stability, and postoperative outcomes, particularly in osteoporotic bone [5,6].

Proximal humeral fractures are among the most common osteoporotic fractures in the elderly population and exhibit a continuously rising incidence due to demographic aging [7,8,9,10]. Clinical outcome following operative treatment of these fractures is strongly dependent on local bone quality of the humeral head, as insufficient trabecular support may result in screw loosening, cut-out, or loss of reduction despite modern locking plate technology [11,12,13,14]. Consequently, accurate preoperative assessment of proximal humeral bone quality is highly relevant for treatment planning and implant selection.

Dual-energy X-ray absorptiometry (DXA) is widely accepted as the clinical gold standard for diagnosing osteoporosis and stratifying fracture risk [15,16,17,18]. However, DXA measurements are limited to predefined anatomical regions such as the lumbar spine, proximal femur, and distal forearm and do not provide site-specific information for the proximal humerus [19,20]. Furthermore, DXA is a two-dimensional projection technique and lacks sensitivity to local variations in trabecular microarchitecture, which are critical for implant anchorage [21].

Computed tomography (CT) is routinely performed in the diagnostic workup of proximal humeral fractures and preoperative planning. CT-derived Hounsfield units (HU) reflect X-ray attenuation properties and have been shown to correlate with bone mineral density [22]. This has prompted increasing interest in exploiting clinically available CT data for opportunistic bone quality assessment without additional radiation exposure or imaging procedures. Quantitative computed tomography (QCT) protocols use defined trabecular ROIs and phantom-based HU calibration to derive volumetric BMD (vBMD), and multiple studies have applied this concept to the proximal humerus. In the present study, we compare three CT-based strategies; importantly, the CT-OAM-inspired component is not intended to replicate classical surface-based CT-osteoabsorptiometry mapping of subchondral mineralization (“loading fingerprints”), but instead provides a simplified, ROI-based mean attenuation index for specimen-level comparison.

Krappinger et al. introduced a standardized HU-based calibration method to estimate cancellous BMD of the proximal humerus using clinical CT data and an external calibration phantom [23]. Their approach demonstrated excellent intra- and interobserver reliability and provided clinically interpretable absolute BMD values. However, HU-based calibration approaches are inherently sensitive to scanner settings, reconstruction parameters, and marrow composition, particularly in elderly patients with increased fatty marrow content.

In parallel, CT osteoabsorptiometry (CT-OAM), originally described by Müller-Gerbl and colleagues, represents a distinct analytical concept that focuses on the spatial distribution of subchondral bone mineralization rather than absolute density quantification [24]. Classical CT-OAM is typically applied to subchondral bone plates to derive surface-based density patterns interpreted as long-term “loading fingerprints” of joint surfaces. Importantly, this concept relies on relative HU distributions across a surface rather than on calibration-based absolute mineral density values. While our approach similarly uses an MIP, the subsequent aggregation into a specimen-level mean, without spatial pattern analysis, distinguishes it from classical CT-OAM. Accordingly, the term “CT-OAM-inspired” refers to an ROI-based densitometry readout (mean index) rather than classical subchondral surface mapping. In the present study, we used a CT-OAM-inspired densitometry approach to compute a specimen-level mean index from a standardized central trabecular ROI (excluding cortical bone). No spatially resolved mapping or subchondral bone plate analysis was performed.

More recently, advances in image analysis and segmentation algorithms have enabled quantitative assessment of bone microarchitecture using CT and micro-computed tomography (micro-CT) datasets. Micro-CT is considered the reference standard for three-dimensional evaluation of trabecular architecture and provides highly detailed structural parameters that correlate strongly with mechanical competence [25]. While micro-CT is restricted to ex vivo or small-animal applications due to radiation dose, it enables validation of CT-based workflows and assessment of their translational potential.

Despite the availability of multiple CT-based approaches for assessing bone quality, direct comparisons between HU-calibrated BMD measurements, CT-OAM-derived indices, and quantitative bone microarchitecture workflows across clinical CT and micro-CT modalities remain scarce. In particular, the extent to which relative CT-OAM indices and microarchitecture-based density metrics are transferable across imaging resolutions has not been systematically evaluated in the proximal humerus.

Therefore, this study aimed to systematically compare three CT-based approaches for assessing proximal humeral bone quality: (i) a HU-based calibration method according to Krappinger et al., (ii) a CT-OAM-inspired workflow yielding a relative density index reported in arbitrary units (a.u.), and (iii) a quantitative Bone Microarchitecture (BMA) workflow implemented in Analyze 15.0. For clarity, (ii) represents a simplified ROI-mean attenuation index and does not produce classical CT-OAM subchondral mineralization maps. By applying these methods to paired clinical CT and micro-CT datasets of human proximal humeral heads, we sought to evaluate their agreement, modality transferability, and potential relevance for patient-specific, site-specific stratification of proximal humeral bone quality in the preoperative setting.

## 2. Materials and Methods

This work was designed as an experimental, ex vivo cross-sectional study comparing three approaches for quantifying bone density-related characteristics of the proximal humeral head using paired clinical CT and micro-CT datasets. All analyses were performed at the specimen level (proximal humeral head). Although bilateral specimens were available from individual donors, the study was designed as a methodological comparison without population-level inference. Twelve proximal humeral heads from six body donors (age 65–86 years; both sides per donor) were provided by the Anatomical Institute of the Medical University of Innsbruck. Because specimens were bilateral (two per donor), observations were not fully independent; wherever applicable, inferential comparisons were treated as paired within donors, and results should be interpreted as methodological rather than population-level inference. Since exclusively body donor specimens were analyzed and no patient-identifiable information was processed, an ethics committee vote was not required.

### 2.1. Imaging Acquisition and Data Export

Clinical CT datasets were acquired on a multidetector CT system (LightSpeed VCT; GE Healthcare, Milwaukee, WI, USA) and exported in Digital Imaging and Communications in Medicine (DICOM) format for downstream post-processing. Micro-CT datasets were acquired using a CT system from SCANCO Medical AG (Brüttisellen, Switzerland). Micro-CT scans were performed at a tube voltage of 59.4 kVp, tube current of 200 µA, and integration time of 180 ms, with threefold projection averaging. The reconstructed isotropic voxel size was 0.082 mm × 0.082 mm × 0.082 mm. Image volumes comprised 758 × 758 pixels per slice with a dataset-dependent number of slices (typically ~700–720). Factory calibration against a 200 mg hydroxyapatite (HA) phantom wedge was applied (60 kVp, 200 mg HA wedge, 8192 scaling), resulting in density values in mg HA/cm^3^. Data were exported in DICOM format; linear attenuation coefficients were stored in [1/cm]. Acquisition parameters and quality assurance procedures are summarized in Table 1.

### 2.2. Software and Computing Environment

All image processing and quantitative analyses were performed using Analyze (Analyze 15.0; AnalyzeDirect, Mayo Clinic, Rochester, MN, USA). For CT-OAM index extraction and automated grayscale statistics, MATLAB (MATLAB R2024a Update 4; The MathWorks Inc., Natick, MA, USA) was used. Image cropping for densitogram standardization was performed in Microsoft PowerPoint 2016 (Microsoft Corporation, Redmond, WA, USA), and tabular post-processing was performed in Microsoft Excel 2016 (Microsoft Corporation). Statistical analysis was conducted using IBM SPSS Statistics (Version 30.0.0; IBM Corp., Armonk, NY, USA).

### 2.3. Image Pre-Processing and Anatomical Standardization

**Figure 1:**
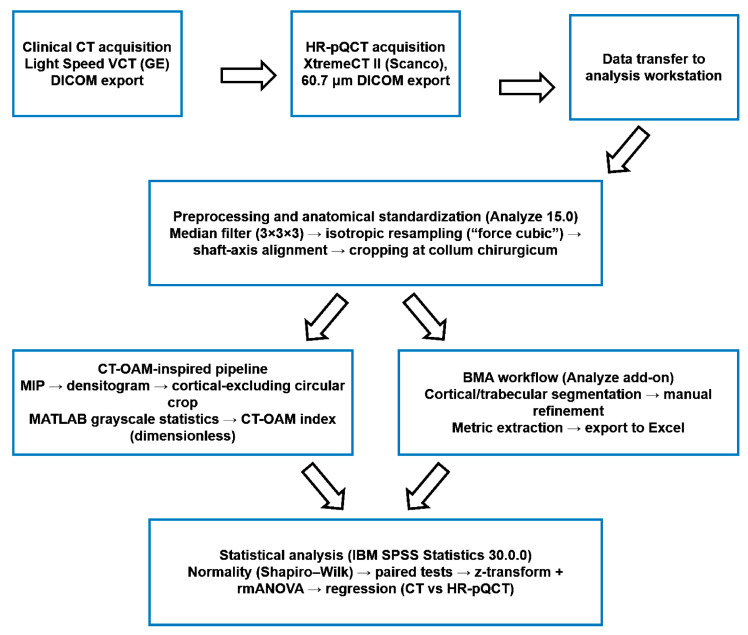
Overview of imaging, pre-processing, and quantitative analysis workflow.

Micro-CT datasets were imported into Analyze 15.0 via the Input function and denoised using a 3D median filter with a kernel size of 3 in all axes (3 × 3 × 3 voxels). To ensure compatibility with downstream tools and to standardize spatial sampling, voxels were resized to cubic geometry using the “force cubic” option; for micro-CT data, the voxel size was additionally set to 0.2 mm to obtain a dataset size manageable for the hardware and software environment while preserving relevant trabecular information. We acknowledge that this resampling step reduces spatial sampling relative to the native 0.082 mm voxel size and may attenuate fine trabecular detail (e.g., thin trabeculae), increase partial-volume averaging, and influence segmentation-derived microarchitectural indices. Accordingly, micro-CT-based morphometry in this study should be interpreted in the context of the resampled resolution, and comparisons focus on methodological associations rather than absolute equivalence of microarchitectural parameters. Voxel size was harmonized to 0.2 mm to balance computational feasibility and the preservation of relevant trabecular features, rather than to enforce artificial similarity between CT and micro-CT modalities. To standardize anatomical orientation, the humeral head volumes were aligned in Analyze using Transform → Apply Matrix, employing a perpendicular axis reference to obtain a consistent orthogonal orientation across specimens. Volumes were then cropped distally using Transform → Subregion, with the surgical neck used as the distal landmark for defining the analysis extent. For clinical CT datasets, voxel resizing was limited to the “force cubic” setting without further resolution changes, as the datasets were already sufficiently small for processing. In clinical CT datasets containing both upper limbs, the two humeri were separated using Transform → Subregion before applying the identical subsequent workflow steps. A schematic overview of the complete image-processing and analysis pipeline is provided in Figure 1.

### 2.4. CT-OAM-Inspired Workflow and CT-OAM Index Computation

A workflow inspired by CT osteoabsorptiometry (CT-OAM) was implemented to derive a specimen-level, ROI-averaged attenuation index and not an absolute BMD value. Importantly, in the present study, we do not compute spatially resolved subchondral mineralization “maps”; instead, we reduce the output to a mean-intensity index measured in a standardized central trabecular ROI (cortical bone excluded). Accordingly, the reported CT-OAM index represents an ROI-mean grayscale/HU-scaled summary and does not quantify the subchondral bone plate or loading-related density distribution patterns in the classical CT-OAM sense.

After standardized orientation, the axial extent of the humeral head (from the proximal first slice showing bony structures to the distal surgical neck landmark) was determined. The total number of slices spanning this region was divided by five to define reproducible measurement locations, and the analysis was performed on the three central slice levels. To reduce sensitivity to single-slice noise and standardize the representation of dense structures, maximum-intensity projections (MIPs) were generated at these levels prior to ROI extraction. For micro-CT datasets, MIPs were generated over seven slices (thickness 1.049 mm; Thick Type = MIP). For clinical CT datasets, MIPs were generated over two slices (slice spacing 1.278 mm). These MIP settings were used to standardize the inputs for ROI-based index computation and visualization.

**Figure 2:**
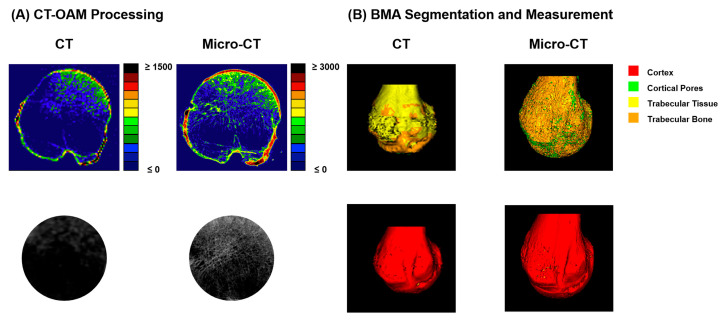
Representative CT-OAM and BMA outputs used for quantitative analysis.

For visualization/densitogram depiction, the HU display range was set to 0–3000 HU for micro-CT and 0–1500 HU for clinical CT. These window settings were applied for visualization only; quantitative calculations were performed on the reconstructed voxel/grayscale values used to generate the MIP images. Representative outputs and the standardized processing steps used for index computation are illustrated in Figure 2.

For each selected slice level, a circular ROI was cropped from the center of the humeral head under the explicit constraint that cortical bone was excluded. Because humeral head size varied between specimens, the ROI radius was adapted per specimen to ensure consistent cancellous sampling while avoiding cortical inclusion. The resulting ROI images were analyzed using a MATLAB script that extracted all non-black pixels and computed descriptive grayscale statistics (minimum, maximum, quartiles, median, mean, standard deviation). The CT-OAM index was defined as the mean ROI grayscale value normalized to the available 8-bit range and scaled by the modality-specific maximum HU window used for densitogram depiction, computed as:

CT-OAM Index (CT) = (Mean/256) × 1500

CT-OAM Index (micro-CT) = (Mean/256) × 3000

For each specimen, the mean of the three slice-based CT-OAM index values was used as the final CT-OAM index for method comparison. Thus, the CT-OAM output in this study is a single specimen-level index reflecting mean cancellous attenuation at standardized humeral head levels, facilitating comparison with HU-calibrated vBMD and BMA-derived metrics.

### 2.5. Bone Microarchitecture (BMA) Workflow in Analyze 15.0

Key analysis settings for CT-OAM and the BMA workflow, including modality-specific thresholds and scaling, are listed in Table 2. Quantitative structural analysis was performed using the Bone Microarchitecture (BMA) add-on implemented in Analyze 15.0. The BMA workflow followed the four-stage structure described in the thesis, comprising pre-processing, cortical segmentation, trabecular segmentation, and measurement. For micro-CT datasets, cortical segmentation was initiated with a lower threshold (Min) of 600, with the upper threshold determined automatically by the software, and trabecular segmentation used a lower threshold (Min) of 200, with the upper threshold determined automatically. For clinical CT datasets, cortical segmentation used a lower threshold of 300, and trabecular segmentation used a lower threshold of 200, with the respective upper thresholds automatically determined. Because automated segmentation did not reliably separate cortical and trabecular components in all slices, manual refinement was performed on the object maps. The automatically generated object map was saved, reopened in the Segment tool, and corrected slice-by-slice in the axial view; cortical contours were refined using Free Hand Draw, while trabecular compartments were corrected using Nudge Edit, after which the edited object map was saved and imported back into the BMA add-on for final measurement extraction. Output parameters were exported to an Excel file for subsequent statistical analysis. For CT-based BMA measurements requiring absolute density scaling, the thesis describes a calibration step based on defining cylindrical reference regions in the CT images, extracting mean grayscale values for each reference insert, and performing linear regression in Excel between grayscale values and known reference densities to derive calibration parameters (SigmaCT as slope and BetaCT as intercept). These parameters were then entered in the BMA add-on, under the BMD scaling parameter settings, to convert grayscale values to BMD (mg/cm^3^) for the calibrated CT-based BMA workflow.

### 2.6. HU-Based Calibrated BMD According to Krappinger et al.

For reference comparison, BMD values previously obtained using the standardized HU-based method described by Krappinger et al. were incorporated into the analysis, as explicitly stated in the thesis. In brief, the Krappinger method defines true axial humeral head planes using multiplanar reconstruction and measures cancellous HU values within three equidistant axial ROIs, with ROI diameters reduced by 15% to exclude cortical bone. HU values are corrected for values below the water equivalent of the European Forearm Phantom, and a linear calibration equation derived from phantom reference sections converts HU to BMD (mg/cm^3^). Accordingly, this HU-to-BMD conversion represents a phantom-calibrated single-energy QCT-style measurement of cancellous vBMD and serves as the clinically most established CT-based reference approach in our comparison.

### 2.7. Statistical Analysis

Statistical analyses were performed in IBM SPSS Statistics (Version 30.0.0; IBM Corp., Armonk, NY, USA). Descriptive statistics were computed for all measurements (*n* = 12 humeral heads). The thesis reports that normality assumptions were evaluated using Shapiro–Wilk tests for single variables and for difference scores in paired testing. Comparisons between calibrated BMD values derived from the Krappinger method and the calibrated BMA workflow on CT datasets were performed using paired t-tests. To compare the three CT-based approaches despite differing measurement scales (mg/cm^3^ for Krappinger and BMA versus a CT-OAM index reported in arbitrary units (a.u.)), z-transformation was applied prior to repeated-measures ANOVA; sphericity was assessed using Mauchly’s test, and Greenhouse–Geisser correction was applied where indicated. Linear regression analyses were used to quantify relationships between CT and micro-CT results for the CT-OAM index and BMA outputs, and the thesis documents that the assumptions (linearity, residual normality, homoscedasticity, independence) were checked, including the Durbin–Watson statistic. Statistical significance was defined as *p* < 0.05.

## 3. Results

### 3.1. Study Cohort and Specimen Characteristics

A total of six body donors were included, each contributing both humeri, resulting in 12 proximal humeral heads available for paired analysis across imaging modalities. The cohort comprised four females (66.7%) and two males (33.3%). The overall mean age was 74.6 years; age information was unavailable for one female donor. Among female donors, the mean age was 75 years (range 68–86 years), and among male donors, the mean age was 74 years (range 65–83 years). These donor and specimen characteristics are summarized in Table 3. Because specimens were bilateral (two per donor), results are presented at the specimen level but should be interpreted as methodological findings within this dataset rather than population-level inference.

### 3.2. Descriptive Results of Density-Related Measures

Descriptive statistics were calculated for all quantitative outputs (N = 12 humeral heads) across the evaluated approaches on clinical CT and micro-CT datasets (Table 2). On clinical CT, the Krappinger method yielded a mean trabecular BMD of 114.37 ± 35.13 mg/cm^3^, with a range of 67.33–185.07 mg/cm^3^. In contrast, the BMA workflow applied to the same CT datasets produced higher absolute density values (207.37 ± 23.78 mg/cm^3^; range 177.55–256.24 mg/cm^3^). The CT-OAM-derived metric is reported as a CT-OAM index (a.u.), i.e., an ROI-mean attenuation proxy derived from the standardized central cancellous ROI (cortex excluded) and averaged across three central levels. On clinical CT, the CT-OAM index averaged 166.94 ± 40.12 a.u. (range 104.71–232.99 a.u.) (Table 4). Because the CT-OAM index is scaled using modality-specific maximum attenuation settings, absolute values should not be compared between clinical CT and micro-CT; instead, the index is used for within-modality dispersion and cross-modality association analyses. Accordingly, descriptive comparisons are provided to characterize within-method distributions in this cohort and are not intended to establish clinical thresholds.

When applied to micro-CT datasets, the BMA workflow produced markedly higher absolute values than clinical CT (mean 469.34 ± 30.99 mg/cm^3^; range 408.43–522.82 mg/cm^3^).

This systematic offset is expected and likely reflects a combination of modality-specific calibration (energy spectrum/phantom mapping) and reduced partial-volume averaging at higher spatial resolution. Therefore, absolute BMA values derived from micro-CT and clinical CT should not be interpreted as directly interchangeable. The CT-OAM index on micro-CT likewise exhibited higher values and a broader spread (mean 455.89 ± 132.63 a.u.; range 212.49–635.55 a.u.) (Table 4).

**Figure 3:**
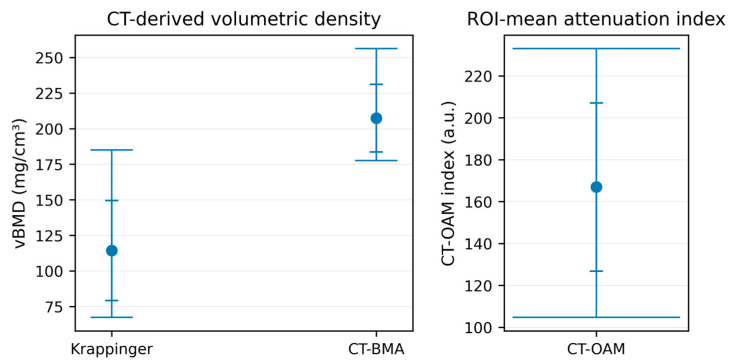
Boxplots of measurement methods derived from clinical CT images. The CT-OAM index is reported in arbitrary units (a.u.) (computed as the mean ROI grayscale intensity normalized to 8-bit range and scaled by the modality-specific maximum attenuation setting), whereas CT-BMA and the Krappinger method report volumetric bone mineral density (vBMD) in mg/cm^3^. Because the methods use different units/scales (a.u. vs. mg/cm^3^), absolute values are not directly comparable across methods; the plot illustrates within-method distributions.

**Figure 4:**
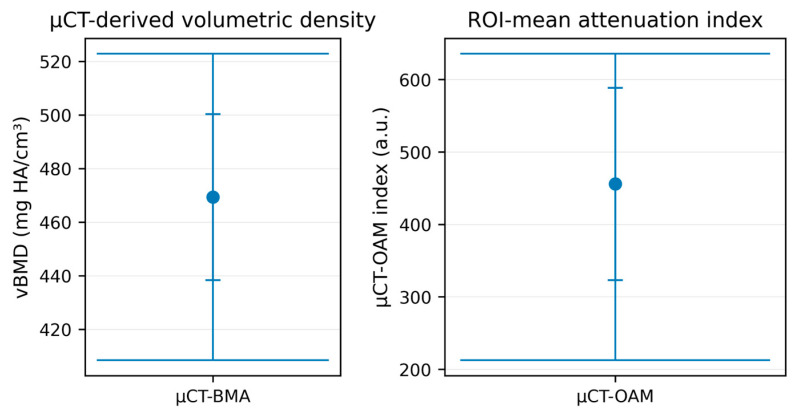
Boxplots of measurement methods derived from micro-computed tomography (micro-CT; µCT) images. The µCT-OAM index is reported in arbitrary units (a.u.), while the µCT-BMA tool reports volumetric bone mineral density (vBMD) in mg HA/cm^3^. Because the methods use different units/scales (a.u. vs. mg HA/cm^3^), absolute values are not directly comparable across methods; the plot illustrates within-method distributions. The µCT-OAM index represents an ROI-mean attenuation proxy and is not a spatial mineralization map.

The distribution of the measurements is summarized visually using boxplots. For the CT-based analyses, the boxplot panel shows the median, interquartile range, and full range (min–max) across the three CT-based approaches (Krappinger BMD, BMA-CT BMD, and CT-OAM index), enabling direct comparison of central tendency and dispersion despite differing output scales (Figure 3). For the micro-CT analyses, the corresponding boxplot panel presents BMA-micro-CT and CT-OAM-micro-CT results and highlights the comparatively wider variability of the CT-OAM index on micro-CT (Figure 4). For clarity, CT-OAM is interpreted here as an ROI-mean index rather than a spatial surface-map output.

### 3.3. Cross-Modality Agreement Between Clinical CT and Micro-CT

To evaluate whether the two newly implemented analysis pipelines provide consistent readouts when applied to different imaging resolutions, we tested the agreement between measurements derived from conventional clinical CT and micro-CT for (i) the CT-OAM index (ROI-mean attenuation proxy) and (ii) the BMA-based density metric. Linear regression analyses were performed with micro-CT as the dependent variable and CT as the predictor, and the standard model assumptions (linearity, normality of residuals, homoscedasticity, and independence) were assessed. The regression results are summarized in Table 5. Given the small sample size (*n* = 12) and bilateral clustering within donors, these regressions are interpreted as descriptive method-agreement analyses within this dataset rather than as population-level predictive models.

**Figure 5:**
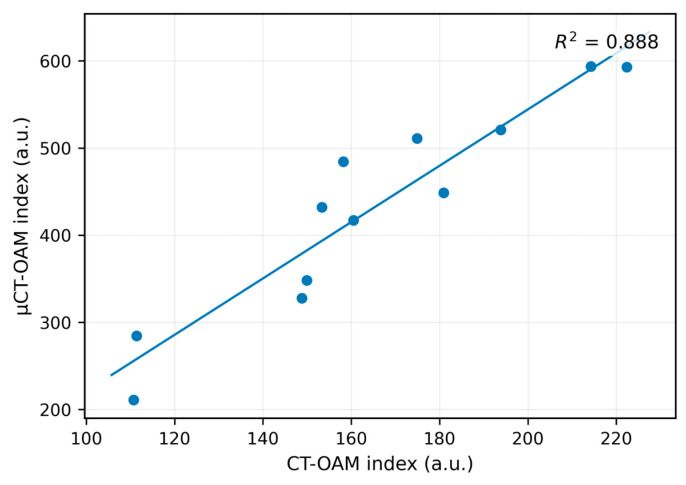
Linear regression between CT-derived CT-OAM index and micro-CT-derived CT-OAM index (*n* = 12). The regression model was significant (F(1, 10) = 79.50, *p* < 0.001) with a high coefficient of determination (R^2^ = 0.888), indicating strong cross-modality agreement of CT-OAM index (ROI-mean attenuation proxy). This reflects within-dataset association and, on its own, does not establish interchangeability across acquisition settings or scanners.

In the CT-OAM approach, CT- and micro-CT-derived indices showed a strong positive association (Figure 5). The regression model was highly significant (F(1, 10) = 79.50, *p* < 0.001) and explained a large proportion of variance (R^2^ = 0.888). The slope was significant (b = 3.12; t = 8.92, *p* < 0.001), whereas the intercept was not (b = −64.19, *p* = 0.309), indicating that specimens with higher CT-derived CT-OAM indices also tended to show proportionally higher micro-CT-derived indices. Residual diagnostics supported model validity (Shapiro–Wilk *p* = 0.578; Durbin–Watson = 1.34). Together, these findings support robust cross-modality rank-order consistency of the CT-OAM index, i.e., an ROI-mean attenuation proxy, across clinical CT and micro-CT. However, statistical significance here should be interpreted in the context of the limited cohort size; the results indicate a strong within-dataset association rather than demonstrating interchangeability or clinical validity.

**Figure 6:**
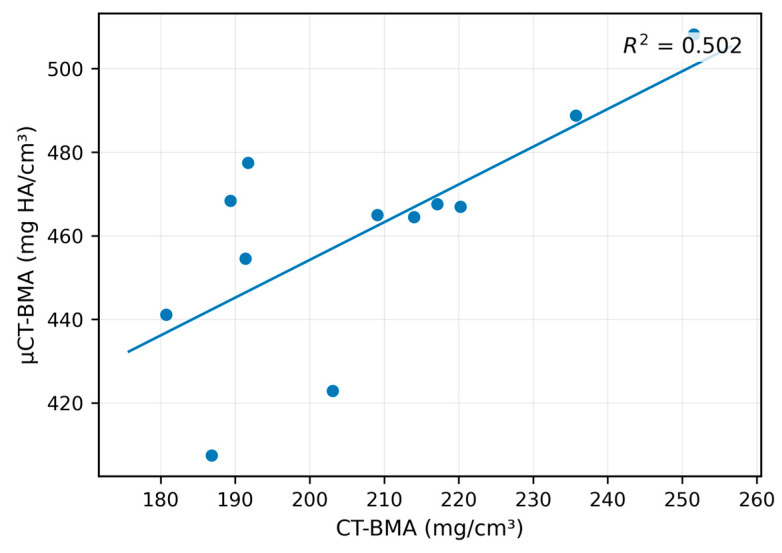
Linear regression between CT-derived BMA measurements and micro-CT-derived BMA measurements (*n* = 12). The regression model was significant (F(1, 10) = 10.06, *p* = 0.010) with moderate explained variance (R^2^ = 0.502). This indicates a moderate linear relationship between modalities but does not demonstrate interchangeability or direct transferability. The observed variance suggests greater dependence on spatial resolution than the CT-OAM-derived index. In the present dataset, statistical significance indicates the presence of a relationship, whereas the effect size (R^2^) reflects its strength; conclusions are therefore based primarily on the magnitude and consistency of the association rather than *p*-values alone.

In the BMA workflow, CT- and micro-CT-based measurements also showed a statistically significant relationship, but the strength of association was lower than for CT-OAM (Figure 6). The regression model remained significant (F(1, 10) = 10.06, *p* = 0.010) with a moderate explained variance (R^2^ = 0.502). Assumption checks were reported as fulfilled, including independence of residuals (Durbin–Watson = 2.27). Collectively, these results indicate that both analysis strategies are transferable across imaging modalities, with markedly stronger cross-modality agreement for CT-OAM than for BMA in this dataset (*n* = 12). Given the small sample size, these findings should be viewed as hypothesis-generating and specific to the present acquisition/processing settings. This is consistent with the expectation that an ROI-mean attenuation index (CT-OAM) is less sensitive to resolution-dependent trabecular segmentation and partial-volume effects than microarchitecture-oriented BMA processing.

### 3.4. Agreement Between HU-Calibrated BMD (Krappinger) and CT-Based BMA-Derived Density

**Figure 7:**
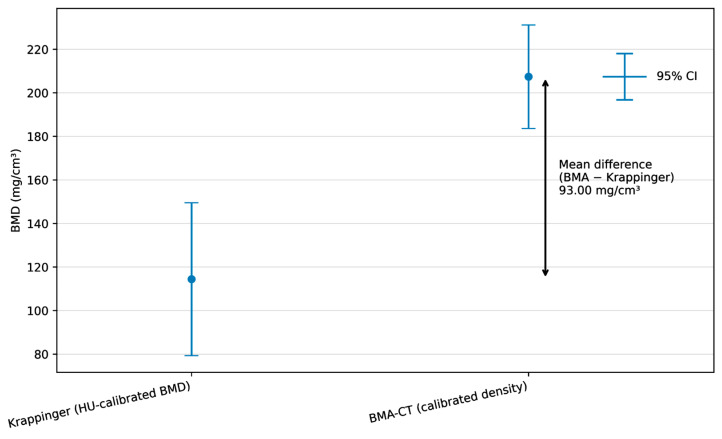
Summary comparison of Krappinger HU-calibrated BMD and CT-based BMA-derived density (*n* = 12). Mean values with standard deviations are shown for the Krappinger method and the BMA workflow applied to clinical CT. The mean paired difference (BMA − Krappinger) and its 95% confidence interval are indicated to visualize the systematic offset between methods.

To determine whether the CT-based BMA workflow yields specimen-level density estimates comparable to those of the established HU-calibrated approach described by Krappinger et al., paired analyses were performed on the clinical CT datasets (*n* = 12). The inferential results, including effect estimates, are summarized in Table 6, and visual agreement is shown in Figure 7.

Normality assumptions were satisfied for both variables and for the paired difference scores (Shapiro–Wilk tests: *p* > 0.05).

In the paired comparison, the BMA-derived CT density values were consistently higher than the Krappinger HU-calibrated BMD values (mean ± SD: 207.37 ± 23.78 mg/cm^3^ vs. 114.37 ± 35.13 mg/cm^3^). The mean paired difference was 93.00 mg/cm^3^, with a 95% confidence interval (CI) of 82.36 to 103.64 mg/cm^3^, indicating a pronounced systematic offset between both approaches. This difference was statistically significant (t(11) = 19.24, *p* < 0.001). Given *n* = 12, interpretation focuses on the magnitude and narrow CI of the paired offset within this cohort, rather than on statistical significance alone.

Despite the systematic level shift, both measures demonstrated a strong positive association across specimens, indicating that individuals with higher HU-calibrated BMD also tended to show higher BMA-derived density values. The correlation between the two methods was high (r = 0.91, *p* < 0.001), supporting rank-order consistency. These findings support within-cohort consistency between methods but do not establish clinical interchangeability or transferable absolute thresholds across scanners/settings.

Effect size metrics indicated a very large standardized difference between methods (Cohen’s d = 5.55; Hedges’ g = 5.17), consistent with the observed magnitude of the paired offset.

### 3.5. Comparison Across the Three CT-Based Approaches After Scale Harmonization

Because the three CT-based approaches yield outputs on different scales and with different physical meanings—absolute density estimates for the Krappinger and BMA workflows (mg/cm^3^) versus a CT-OAM index reported in arbitrary units (a.u.)—direct comparison of raw values is not appropriate. Therefore, as described in the thesis, all CT-derived measures were z-standardized before inferential comparison to remove scale effects and enable evaluation of relative differences in central tendency across methods. The inferential results are summarized in Table 7.

The normality assumptions for the standardized variables were met (Shapiro–Wilk tests: *p* > 0.05). Mauchly’s test indicated violation of sphericity (W = 0.463, *p* = 0.021). Accordingly, Greenhouse–Geisser correction was applied for the repeated-measures ANOVA. Under the Greenhouse–Geisser correction, there was no evidence for differences among the three CT-based approaches (F(1.30, 14.32) = 0.00, *p* = 1.00). These results indicate that, after harmonizing the scale via z-transformation, the three approaches did not differ in their mean standardized outputs across specimens, suggesting comparable relative positioning of samples within the cohort despite differences in absolute units and computational principles. Because of the limited sample size and donor clustering, this result should be interpreted as an absence of detectable differences under z-standardization in this dataset, rather than as evidence of equivalence between methods.

## 4. Discussion

In this study, we investigated site-specific bone quality in the proximal humerus using clinically available CT data complemented by (i) HU-calibrated/QCT-style vBMD, (ii) a CT-OAM-inspired ROI-based attenuation index, and (iii) high-resolution/µCT-derived microarchitectural indices. The overall workflow (Figure 1) was designed to translate routine preoperative imaging into quantitative parameters that may better reflect local trabecular support than systemic surrogates alone. This is clinically relevant because fixation failure after proximal humeral fracture osteosynthesis is frequently driven by local bone insufficiency (e.g., intra-articular screw penetration, varus collapse), even with modern locking plates [26]. Proximal humeral fractures are common in older individuals and are strongly associated with fragility mechanisms. Epidemiologic data consistently show increasing fracture burden with age and a predominance in women; long-term registry and national database analyses demonstrate substantial healthcare impact and evolving treatment trends [27,28,29]. Importantly, complication patterns following locking plate fixation, especially screw penetration and varus collapse, are strongly linked to insufficient osseous support and medial column integrity, underscoring that local bone quality is not a theoretical construct but a major determinant of mechanical success [26]. Biomechanical evidence further indicates that techniques enhancing structural support (e.g., restoring medial column support) improve resistance to varus loading, reinforcing the concept that regional bone competence governs construct stability [30]. Central DXA remains the clinical reference standard for BMD assessment and osteoporosis diagnosis/risk stratification; however, it provides areal BMD at predefined sites and cannot quantify site-specific trabecular microarchitecture relevant for implant anchorage. Contemporary reviews explicitly reiterate DXA as the gold standard while acknowledging the broader concept of skeletal fragility beyond a single metric [31]. This limitation motivates opportunistic imaging strategies that repurpose already-acquired CT scans to obtain bone attenuation metrics. HU from diagnostic CT correlates with bone density and even compressive strength proxies, supporting HU as a pragmatic, low-friction adjunct to systemic assessment [32]. Opportunistic osteoporosis screening using CT attenuation has also been demonstrated at peripheral sites (e.g., wrist CT), underscoring its feasibility within routine workflows without additional radiation exposure [33]. In the proximal humerus, Krappinger et al. specifically evaluated local bone quality and showed that CT-based measures can inform clinically relevant assessment in the humeral head region [23]. Against this background, our approach builds on the premise that regional metrics derived from CT are more directly aligned with the mechanical environment encountered by screws in the humeral head than DXA values from hip/spine. CT-OAM was developed to quantify subchondral mineralization patterns as a marker of long-term joint loading and functional adaptation [24]. In the present work, we explicitly did not apply classical surface-based CT-OAM mapping; instead, we adapted the concept to a simplified trabecular densitometry readout. Specifically, our CT-OAM output is a specimen-level mean index derived from a standardized central cancellous ROI (excluding cortical bone) and averaged across three central levels (Figure 2). Thus, the CT-OAM index should be interpreted as an ROI-mean attenuation proxy that complements HU-calibrated vBMD and µCT-derived microarchitecture, rather than as a measure of subchondral mineralization patterns or articular “loading fingerprints”.

This clarification is important for clinical interpretation. In our workflow, CT-OAM (Figure 2) complements trabecular density/microarchitecture metrics by (i) providing an ROI-averaged attenuation surrogate within the predefined humeral head regions (derived from mean grayscale intensities), and (ii) offering a simple, reproducible index that showed strong cross-modality rank-order consistency in our dataset (Figure 5; R^2^ = 0.888), supporting its robustness across CT and µCT resolution levels. However, because the metric is reduced to a mean-intensity index, it does not capture spatial heterogeneity; any discussion of long-term joint loading and mineralization “fingerprints” therefore serves as background context for classical CT-OAM rather than a direct readout of our adapted index.

Bone strength is governed not only by mineral content but also by microarchitecture (trabecular number, thickness, separation; cortical geometry), which can influence failure mechanisms and implant anchorage. Large prospective cohort work has shown that deficits in cortical and trabecular microarchitecture are associated with fracture risk independently of DXA BMD and FRAX, underscoring the clinical significance of microstructural measures beyond areal BMD [34]. State-of-the-art reviews on Micro-CT describe its ability to quantify microarchitecture in vivo and discuss how these indices can refine fragility phenotyping beyond DXA, albeit with current barriers to widespread clinical adoption [35]. By integrating high-resolution measures into the analysis pipeline (Table 1 and Table 2), the present study aligns with the broader movement from single-parameter bone assessment toward multi-parametric bone quality phenotyping, which is more congruent with the multifactorial causes of fixation failure in osteoporotic bone. A key translational requirement is reproducibility and standardized reporting. For micro-CT-based morphometry, community guidance emphasizes transparent reporting of acquisition parameters, segmentation approach, and a minimal core set of trabecular/cortical outcomes to support comparability across studies [25]. This rationale supports the explicit reporting of scanner settings and reconstruction/analysis parameters (Table 1 and Table 2) and the structured workflow documentation (Figure 1). For Micro-CT, motion artifacts remain a dominant source of variability, and standardized immobilization and quality-control measures are widely recognized as essential [35]. In this context, the combination of daily phantom checks and immobilization strategies is not merely procedural; it directly affects the reliability of microarchitectural indices and, in turn, the interpretability of structure–function relationships. From a clinical decision-support perspective, the most compelling implication is the potential to extract actionable, local bone-quality information from imaging that is already obtained for fracture diagnosis and preoperative planning. Opportunistic HU analysis and region-focused assessment have strong precedent in the literature [23,32,33].

To facilitate clinical translation, a realistic near-term implementation could follow a stepwise roadmap: (i) automated or semi-automated ROI placement on the preoperative CT (aligned with the Krappinger ROIs and the standardized central cancellous ROI used here) to output HU-calibrated vBMD and the CT-OAM ROI-mean index as a short, standardized report; (ii) integration of these outputs into pragmatic preoperative risk stratification (e.g., low/medium/high local trabecular support) to inform surgical planning decisions such as augmentation (cement), calcar/medial support strategies, screw trajectory/length optimization, or implant selection; and (iii) prospective outcome-linked validation to derive clinically meaningful thresholds and to test whether these metrics add predictive value beyond conventional factors (age, fracture pattern, reduction quality, medial hinge integrity, and surgeon-related variables). In this context, the primary deliverable would not be a complex research map but a small set of reproducible, interpretable local bone-quality parameters that can be generated from routine CT without additional radiation or workflow disruption.

The next practical step is to determine whether combined metrics (e.g., HU-calibrated vBMD together with microarchitecture surrogates and/or an ROI-based CT-OAM index) can improve the prediction of fixation-related complications beyond conventional factors (age, fracture pattern, surgeon factors, and systemic osteoporosis status). If classical surface-based CT-OAM mapping is pursued in future work, its added value should be evaluated explicitly against these simpler ROI-based measures, with attention to standardization, interpretability, and clinical workflow integration. In parallel, outcomes-driven validation is necessary: imaging markers should be linked to clinically relevant endpoints such as screw cut-out, head collapse, loss of reduction, and reoperation—events that remain frequent in locking plate cohorts [26].

Several limitations should be considered. First, the sample comprised 12 humeral heads from 6 donors (bilateral), and observations were therefore clustered within donors; although paired testing was applied where appropriate, residual within-donor correlation may affect inferential precision and limit population-level generalizability. In addition, the donor cohort was exclusively elderly (65–86 years); therefore, the observed density and microarchitecture relationships may differ in younger individuals. Second, the CT-OAM approach was intentionally reduced to an ROI-mean index (averaged across slices), thereby omitting assessment of the spatial heterogeneity of subchondral mineralization (“loading fingerprints”). Third, because the CT-OAM index is based on modality-specific scaling (a.u.), it is best suited for within-method comparisons and association analyses rather than as an absolute, interchangeable density metric.

## 5. Conclusions

In this ex vivo study of 12 proximal humeral heads, clinically available CT data could be leveraged to derive site-specific, quantitative measures of local bone quality using both an HU-calibrated reference approach and two complementary image-analysis pipelines (CT-OAM-inspired densitometry and a bone microarchitecture workflow). The HU-based calibration arm of our analysis follows the established proximal-humerus QCT concept (ROI-based HU measurement with phantom-derived linear calibration) as described by Krappinger et al., yielding a clinically interpretable vBMD metric. After scale harmonization, the three CT-based approaches showed no statistically detectable differences in standardized outputs, indicating comparable relative stratification of specimens despite differing units and computational principles. Cross-modality analyses revealed a strong linear association between CT- and micro-CT-derived CT-OAM indices, indicating high relative consistency and stable rank ordering of specimens across modalities rather than direct interchangeability or transferability. In contrast, the BMA workflow demonstrated a significant but weaker association, which may reflect a greater sensitivity of segmentation-based microarchitectural measures to spatial resolution. Finally, although HU-calibrated BMD (Krappinger) and CT-based BMA-derived density were strongly correlated, a pronounced systematic offset was observed, underscoring that absolute values from different calibration frameworks should not be used interchangeably without method-specific harmonization. Overall, these findings support the feasibility of extracting potentially clinically relevant, site-specific bone-quality information from routine CT imaging for the proximal humerus, while acknowledging that the present ex vivo dataset is intended for methodological comparison rather than clinical prediction. Future work should focus on prospective clinical validation in adequately powered cohorts linking these quantitative markers to fixation-related outcomes (e.g., screw loosening or cut-out) and on establishing robust, scanner-independent standardization to enable broader clinical translation.

## Figures and Tables

**Table 1:** Imaging acquisition and quality assurance parameters.

Modality	Parameter	Value
Clinical CT	Scanner	LightSpeed VCT
Clinical CT	Export format	DICOM
Micro-CT	System	CT system; Software version SCANCO_V1.1c
Micro-CT	Modality (DICOM)	CT
Micro-CT	Tube voltage	59.4 kVp
Micro-CT	Tube current	200 µA
Micro-CT	Integration time	180 ms
Micro-CT	Projection averaging	3
Micro-CT	Reconstructed voxel size	0.082 mm isotropic
Micro-CT	In-plane matrix	758 × 758 pixels per slice
Micro-CT	Number of slices	Dataset-dependent (typically ~700–720)
Micro-CT	Calibration	Factory calibration vs. 200 mg HA phantom wedge
Micro-CT	Density units	mg HA/cm^3^
Micro-CT	Stored quantity	Linear attenuation coefficients [1/cm]
Micro-CT	Export format	DICOM

**Table 2:** Core analysis parameters for CT-OAM and BMA workflows.

Workflow	Setting	Value
CT-OAM	MIP thickness	Clinical CT: 2 slices; Micro-CT: 7 slices
CT-OAM	HU display range (densitograms)	Clinical CT: 0–1500 HU; Micro-CT: 0–3000 HU
CT-OAM	ROI standardization	Central circular crop excluding cortical bone
CT-OAM	Index computation	Index = (Mean grayscale/256) × HUmax; mean across selected slices
BMA	Software	Analyze 15.0; Bone Microarchitecture add-on
BMA	Thresholding (lower bounds)	CT cortical Min 300; CT trabecular Min 200; Micro-CT cortical Min 600; Micro-CT trabecular Min 200
BMA	Upper bounds	Automatically determined by software
BMA	Manual refinement	Slice-wise editing of object maps (Free Hand Draw/Nudge Edit)
BMA	Density scaling (CT)	Linear regression-derived slope/intercept entered into BMD scaling settings
Statistics	Software	IBM SPSS Statistics 30.0.0

**Table 3:** Donor and specimen characteristics.

Parameter	Total Cohort	Female Donors	Male Donors
Donors (*n*)	6	4	2
Specimens (humeral heads, *n*)	12 (bilateral; 2 per donor)	8	4
Sex distribution	66.7% female/33.3% male	—	—
Age, mean (years)	74.6	75	74
Age, range (years)	—	68–86	65–83

**Table 4:** Descriptive statistics of density-related measures.

Modality	Method/Output	Unit	*n*	Mean	SD	Min	Max
Clinical CT	Krappinger HU-calibrated cancellous BMD	mg/cm^3^	12	114.37	35.13	67.33	185.07
Clinical CT	BMA workflow (calibrated density)	mg/cm^3^	12	207.37	23.78	177.55	256.24
Clinical CT	CT-OAM index	a.u.	12	166.94	40.12	104.71	232.99
Micro-CT (SCANCO)	BMA workflow	mg HA/cm^3^ *	12	469.34	30.99	408.43	522.82
Micro-CT (SCANCO)	CT-OAM index	a.u.	12	455.89	132.63	212.49	635.55

* Micro-CT values are reported in mg HA/cm^3^ based on the acquisition calibration described in the DICOM metadata.

**Table 5:** Regression summary for cross-modality agreement.

Outcome (Micro-CT)	Predictor (CT)	*n*	F (df1, df2)	*p*-Value	R^2^	Slope b (Significance)	Intercept (Significance)
CT-OAM index (micro-CT)	CT-OAM index (CT)	12	79.50 (1, 10)	<0.001	0.888	b = 3.12; t = 8.92, *p* < 0.001	b = −64.19, *p* = 0.309
BMA measurement (micro-CT)	BMA measurement (CT)	12	10.06 (1, 10)	0.010	0.502	not reported in excerpt	not reported in excerpt

**Table 6:** Paired comparison between Krappinger HU-calibrated BMD and CT-based BMA-derived density.

Comparison (Clinical CT)	*n*	Mean (Krappinger), mg/cm^3^	Mean (BMA-CT), mg/cm^3^	Mean Difference (BMA − Krappinger), mg/cm^3^	95% CI of Difference, mg/cm^3^	t (df)
BMA-CT vs. Krappinger	12	114.37	207.37	93.00	82.36–103.64	19.24 (11)

**Table 7:** Repeated-measures ANOVA comparing z-standardized outputs of three CT-based approaches (*n* = 12).

Analysis	*n*	Methods Compared	Pre-Processing	Mauchly’s W	*p* (Mauchly)	Correction	F (df1, df2)	*p*-Value
rmANOVA (within-subject)	12	Krappinger BMD vs. BMA-CT density vs. CT-OAM index	z-standardization	0.463	0.021	Greenhouse–Geisser	0.00 (1.30, 14.32)	1.00

## Data Availability

The data presented in this study are available on request from the corresponding author. The data are not publicly available due to scientific reasons.
